# Glycogen myophosphorylase loss causes increased dependence on glucose in iPSC-derived retinal pigment epithelium

**DOI:** 10.1016/j.jbc.2024.107569

**Published:** 2024-07-14

**Authors:** Basudha Basu, Magdalena Karwatka, Becky China, Martin McKibbin, Kamron Khan, Chris F. Inglehearn, John E. Ladbury, Colin A. Johnson

**Affiliations:** 1 Division of Molecular Medicine, Leeds Institute of Medical Research, University of Leeds, Leeds, UK; 2 Department of Ophthalmology, St James’s University Hospital, Leeds, UK; 3 School of Molecular and Cellular Biology, University of Leeds, Leeds, UK

**Keywords:** retina, retinal dystrophy, retinal degeneration, retinal metabolism, glucose metabolism, induced pluripotent stem cell (iPS cell) (iPSC)

## Abstract

Loss of glycogen myophosphorylase (PYGM) expression results in an inability to break down muscle glycogen, leading to McArdle disease—an autosomal recessive metabolic disorder characterized by exercise intolerance and muscle cramps. While previously considered relatively benign, this condition has recently been associated with pattern dystrophy in the retina, accompanied by variable sight impairment, secondary to retinal pigment epithelial (RPE) cell involvement. However, the pathomechanism of this condition remains unclear. In this study, we generated a PYGM-null induced pluripotent stem cell line and differentiated it into mature RPE to examine structural and functional defects, along with metabolite release into apical and basal media. Mutant RPE exhibited normal photoreceptor outer segment phagocytosis but displayed elevated glycogen levels, reduced transepithelial resistance, and increased cytokine secretion across the epithelial layer compared to isogenic WT controls. Additionally, decreased expression of the visual cycle component, RDH11, encoding 11-cis-retinol dehydrogenase, was observed in PYGM-null RPE. While glycolytic flux and oxidative phosphorylation levels in PYGM-null RPE were near normal, the basal oxygen consumption rate was increased. Oxygen consumption rate in response to physiological levels of lactate was significantly greater in WT than PYGM-null RPE. Inefficient lactate utilization by mutant RPE resulted in higher glucose dependence and increased glucose uptake from the apical medium in the presence of lactate, suggesting a reduced capacity to spare glucose for photoreceptor use. Metabolic tracing confirmed slower ^13^C-lactate utilization by PYGM-null RPE. These findings have key implications for retinal health since they likely underlie the vision impairment in individuals with McArdle disease.

McArdle disease is a glycogen storage disease (type V; OMIM #232600) that has an autosomal recessive pattern of inheritance. It is a disorder of glycogen metabolism resulting from mutations in the muscle isoform of glycogen phosphorylase (PYGM) (1, 2, 3). This enzyme catalyzes the first step in the breakdown of glycogen to glucose (Fig. 1*A*), and patients with this mutation are unable to use glycogen as an energy source. While not life-threatening and previously assumed to be relatively benign, this condition causes exercise intolerance, reversible episodes of contractures, rhabdomyolysis, and myoglobinuria, which, in severe cases, can lead to acute renal failure (4). Although McArdle disease was first described in 1951 (5), the association with late-onset retinal dystrophy, in particular, pattern dystrophy of the retinal pigmented epithelium (RPE) is more recent (6, 7, 8, 9, 10). Three isoforms of glycogen phosphorylase exist and, historically, have been named for their tissue-specific expression mainly in the brain (PYGB), liver (PYGL), and muscle (PYGM) (1, 11). However, their expression patterns are now known to be broader, with PYGM expressed in muscles, retina, brain, granulocytes, and adipose tissue and PYGB in the brain, retina, and cardiac muscles (11). PYGL is expressed in the liver and also in a number of tumor types under hypoxic conditions including gliomas (12). Although both PYGB and PYGM are expressed in the RPE (13, 14, 15), it is believed that PYGM mediates retinal glycogenolysis because PYGB is not sufficient for retinal energy metabolism possibly due to its sensitivity to cellular AMP levels (9, 11).

**Figure 1:**
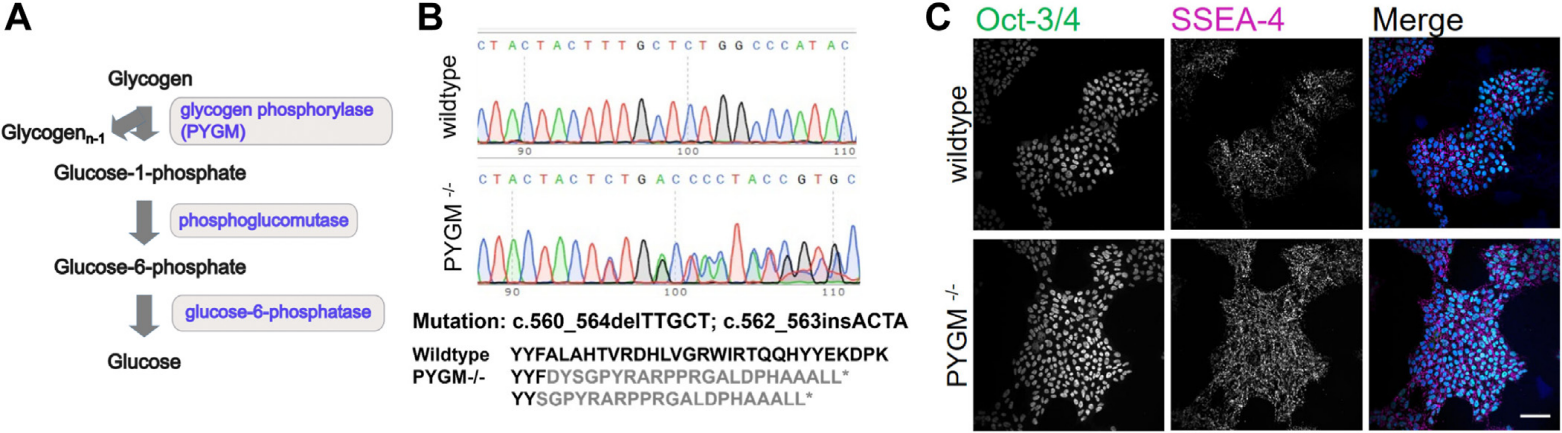
**Generating the PYGM null iPSC mutant line.***A*, the PYGM enzyme is required for the first step in the breakdown of glycogen to glucose. It generates glycogen_n-1_ and glucose-1-phosphate. *B*, sequence trace of the WT and mutant showing the biallelic indel mutation [c.560_564delTTGCT]+[c.562_563insACTA] and the resulting protein sequence. ∗ represents the premature termination codon. *C*, the PYGM^−/−^ null mutant iPSC line expresses the pluripotency markers Oct-3/4 (*green*) and SSEA-4 (*magenta*) with counterstain for DAPI (*blue*) 20×. Scale bar represents 100 μm.

The RPE cells form the outer blood–retina barrier, keeping the eye immunologically privileged. This layer also delivers nutrients from the blood stream to the retina, absorbs scattered light, phagocytoses the shed photoreceptor outer segments (POSs), secretes cytokines, and recycles retinoids for the visual cycle (16, 17). Glucose is the primary energy source for the retina and most of it comes into the retina from the blood stream *via* the RPE cells. However, RPE cells also have stores of glycogen (13) that they can break down, in order to buffer glucose levels for managing the demand of the photoreceptor cells (18). RPE cells suppress their own glucose consumption and, instead, preferentially utilize lactate released by the highly glycolytic photoreceptor cells, allowing more glucose to be supplied to the photoreceptor layer (19, 20).

The ability to introduce mutations in induced pluripotent stem cells (iPSCs) and differentiate them into RPE cells affords a way to model the effects of PYGM loss in these cells. We introduced a null mutation into the *PYGM* gene using CRISPR-Cas9 gene editing, which functionally mimicked the common McArdle null variant, p.R50∗ (21, 22). We subsequently assayed the iPSC-derived RPE cells for structural, functional, and metabolic deficits. We observed defects in several aspects of RPE function, most importantly a reduced ability to use lactate in preference to glucose. Collectively, these findings suggest a pathomechanism for late-onset vision loss in individuals with McArdle disease.

## Results

### Characterizing the PYGM mutant iPSC line

The AD2 WT human iPSC line (23) was subjected to the CRISPR-Cas9 protocol (24) as described in the Experimental procedures and then index-sorted by FACS to obtain single-cells which were allowed to form clonal colonies. On sequencing, we identified one mutant clone, which had biallelic indels in *trans*, causing frameshift mutations. One allele had a 5 bp deletion (c.560_564delTTGCT) and the other a 4 bp insertion (c.562_563insACTA) (Fig. 1*B*) in *PYGM* (transcript reference NM_005609.4, genome build GRCh38), with predicted coding consequence [p.(F54Sfs∗22)]+[p.(A55Dfs∗24)]. Both frameshift mutations were predicted to cause nonsense-mediated decay (NMD) of the *PYGM* transcript, using NMDEscPredictor (25) and Mutation taster (26). Off-target effects of the guide RNA on other genes in the mutant line were excluded by sequencing (Table S2). The *PYGM* mutant iPSC line expressed the pluripotency markers Oct3/4 and SSEA4 with the same pattern and to the same extent as the isogenic-matched WT control line (Fig. 1*C*).

### RPE cells derived from *PYGM* mutant iPS cells do not express PYGM

**Figure 2:**
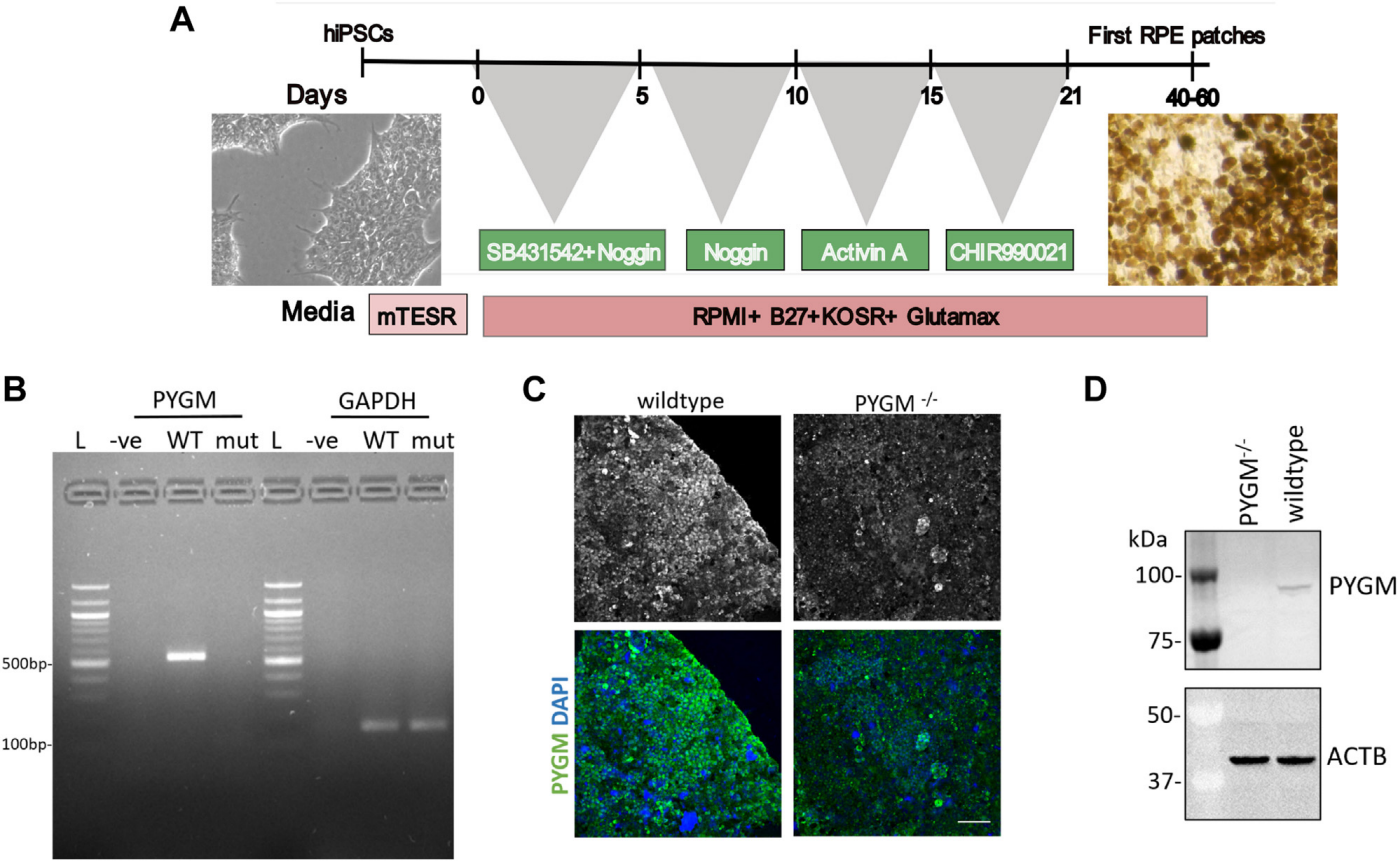
**Differentiated mutant RPE cells do not express PYGM.***A*, schematic of differentiation protocol for generating RPE cells from undifferentiated iPSCs with sequential addition of a series of compounds. *B*, RT-PCR to detect transcripts of PYGM and GAPDH. *C*, antibody staining of WT and *PYGM*^*−/−*^ RPE with an anti-PYGM antibody shows reduced staining in the mutant. Scale bar represents 100 μm. *D*, immunoblot showing the loss of PYGM protein in the mutant. ACTB used as a loading control marker. Full, uncropped immunoblots are shown in Fig. S2*A*. L, 100 bp ladder; mut, PYGM^−/−^ RPE complementary DNA; –ve, negative control; WT, wildtype RPE complementary DNA.

RPE cells were derived from WT isogenic control and *PYGM* mutant iPS cells using a previously established protocol (23). Briefly, iPSCs were treated with a mixture of SB431542 and Noggin for 5 days and then Noggin alone for 5 days. This was followed by 6 days of treatment with activin A, a RPE promoting factor, and then 6 days of treatment with a Wnt agonist, CHIR99021, before allowing the cells to differentiate under the control of intrinsic factors to eventually form patches of RPE (Fig. 2*A*). *PYGM* transcripts were undetectable (Fig. 2*B*) and protein levels were significantly decreased in mutant RPE cells as expected (Fig. 2*C*). Immunoblotting against PYGM confirmed loss of protein in mutant RPE cells (Fig. 2*D*). We excluded potential compensatory upregulation of the other isoforms, *PYGB* and *PYGL*, by confirming that their expression levels were unchanged compared to WT isogenic control RPEs (Fig. S1). The *PYGM* mutant therefore carries two null frameshift alleles in *trans*, and so we designated the genotype for this line as *PYGM*^−/−^.

### Immunolabeling and transcript analysis reveal difference in the levels of *RDH11* transcript between WT and mutant RPE

**Figure 3:**
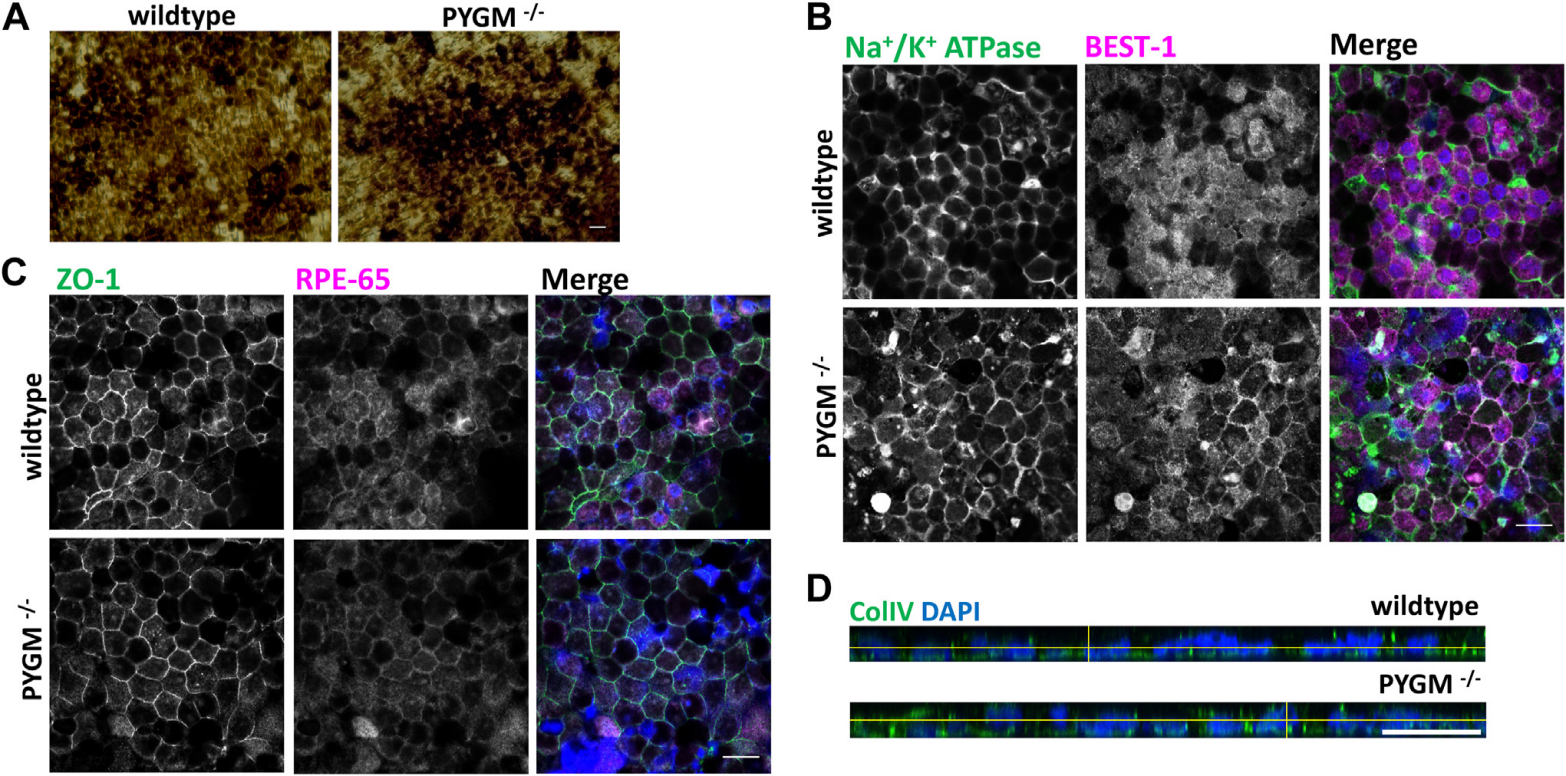
**Immunolabeling showing cellular localization of characteristic RPE markers.***A*, WT and mutant RPE cells showing characteristic cellular morphology and pigmentation. *B*, localization of ZO-1 (*green*) and RPE-65 (*magenta*) in WT and *PYGM*^*−/−*^ mutant RPE cells. *C*, WT and mutant RPE cells stained for Na^+^/K^+^ ATPase (*green*) and BEST-1 (*magenta*). *D*, orthogonal section of RPE cells stained with Collagen IV (*green*) showing basolateral localization. All immunolabeled cells are counterstained with DAPI to mark nuclei (*blue*). Scale bar represents 20 μm.

**Figure 4:**
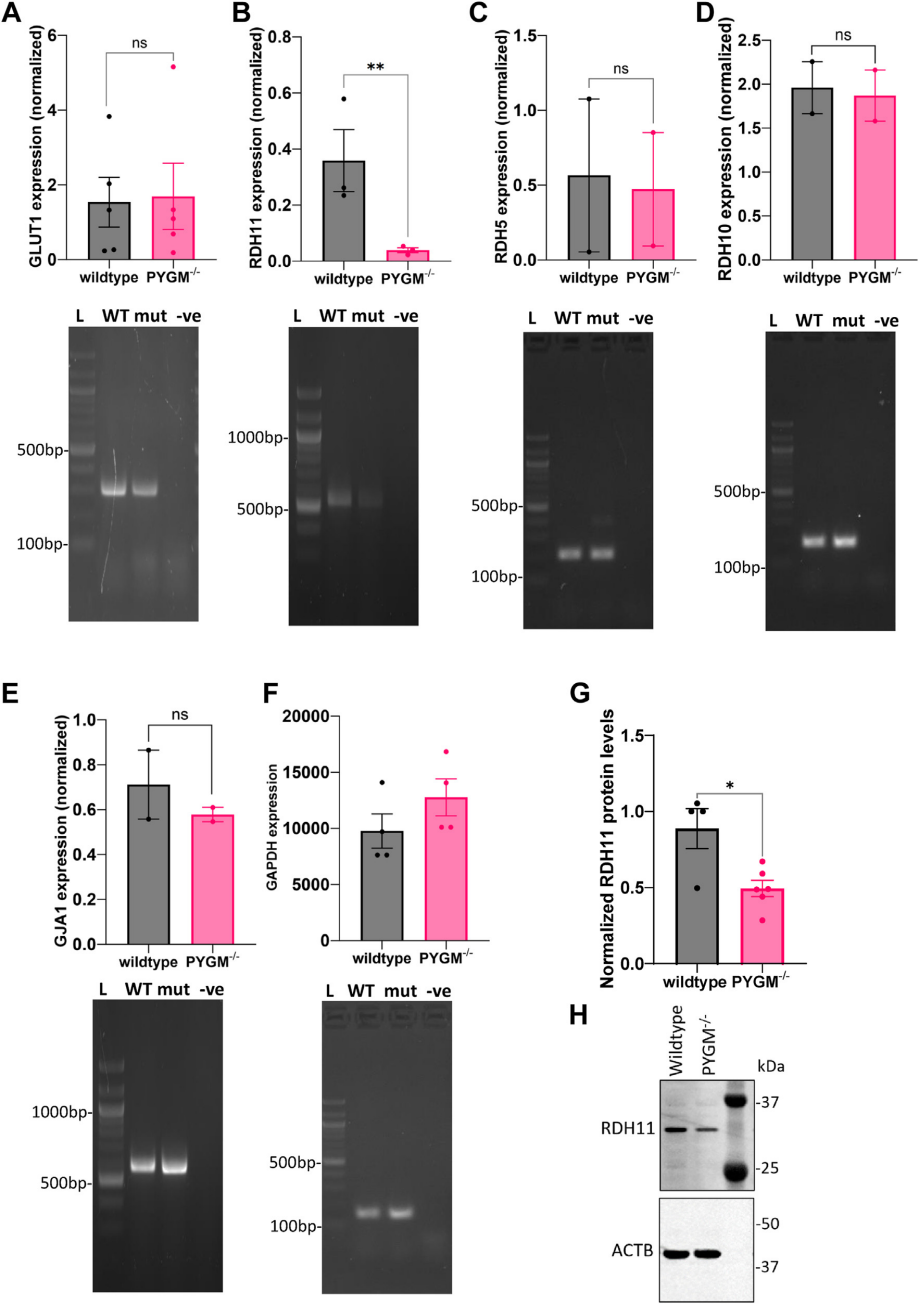
**RT-PCR to compare the expression of key genes in WT and mutant RPE.** Representative gel images of RT-PCR assays of transcript expression for: (*A*) *GLUT1*, (*B*) *RDH11*, (*C*) *RDH5*, (*D*) *RDH10*, (*E*) *GJA1*, (*F*) *GAPDH*. Data points represent biological replicates and the data was normalized to *GAPDH* levels of the samples. Paired Student t-tests were used for pair-wise statistical analyses as indicated. *G* and *H*, immunoblot to show RDH11 protein levels in WT and mutant RPE, with quantification of protein levels normalized to ACTB. Full, uncropped immunoblots are shown in Fig. S2*B*. Data points represent biological replicates. Error bars show SEM. ∗*p* < 0.05, ∗∗*p* < 0.01, ∗∗∗*p* < 0.001, # *p* < 0.0001. Lanes L, ladder; WT, wildtype RPE; mut, PYGM^−/−^ RPE; -ve, negative control.

Both the WT control and mutant RPE had the classical, pigmented, and cobblestone appearance with areas of dark and light pigmentation (Fig. 3*A*). We examined the cellular localization of key RPE markers in WT and mutant cells. Both expressed markers in patterns characteristic of RPE cells, including the ZO-1, RPE-65, Na^+^/K^+^ ATPase, and bestrophin-1 (BEST-1) proteins (Fig. 3, *B* and *C*). The basolateral marker collagen IV (ColIV) appeared correctly localized in both WT and mutant cells (Fig. 3*D*). We used semi-quantitative RT-PCR in WT and mutant RPE to assay the expression of three signature genes comprising a metabolic, structural, and visual cycle gene (*GLUT1*, *GJA1*, and *RDH11*, respectively) (27). *GLUT1* (encoding glucose transporter 1) and *GJA1* (encoding gap junction protein connexin43) did not show significant differences (Fig. 4, *A* and *E*). Contrary to expectations, *GLUT1* was not upregulated in the mutant as a compensation for the inability to breakdown glycogen for glucose. In contrast, the *RDH11* transcript (encoding a 11-*cis*-retinol dehydrogenase) was significantly downregulated in mutant RPE (Fig. 4*B*). However, expression levels of *RDH10* and *RDH5*, both encoding other retinol dehydrogenases of the visual cycle in RPE, were not significantly affected in the mutant (Fig. 4, *C* and *D*). We further examined protein levels of RDH11 using Western blot and found that its expression was, indeed, significantly reduced (Fig. 4, *G* and *H*).

### Phagocytosis is unaffected in mutant RPE cells but they show reduced transepithelial resistance and increased cytokine secretion

**Figure 5:**
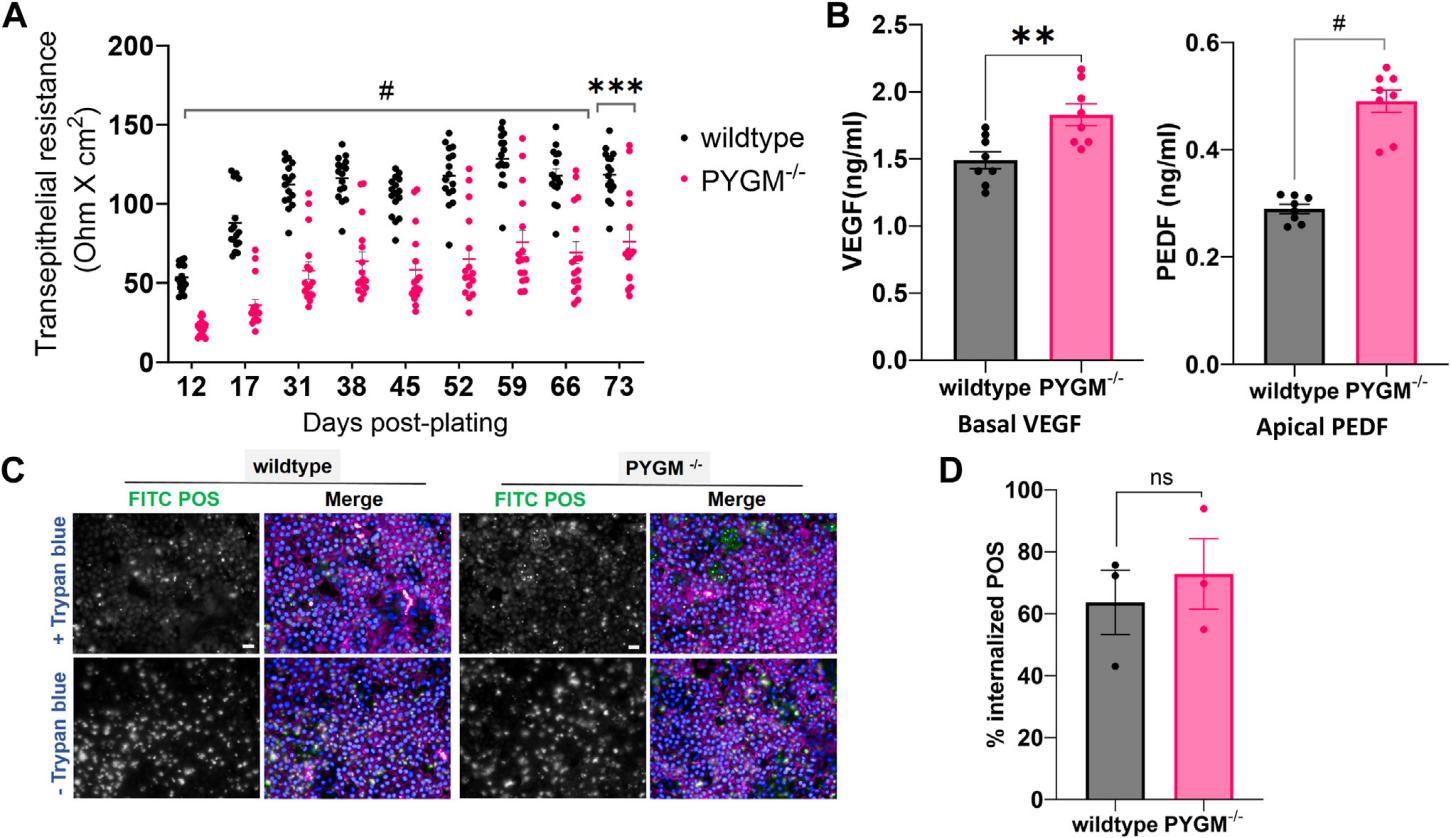
**Examining TER, cytokine secretion, and phagocytosis in WT and *PYGM***^***−/−***^**mutant RPE cells.***A*, transepithelial resistance measured over 77 days in WT and *PYGM*^*−/−*^ RPE cells freshly plated on inserts. Each data point represents an individual insert. Two-way ANOVA followed by pairwise comparison with Sidak’s correction was performed. *B*, measurements of VEGF secreted into basal medium and PEDF secreted apically in WT and *PYGM*^*−/−*^ RPE cells plated on inserts. Data points representing four technical replicates each of two biological replicates are shown. Paired t-tests were used for statistical analyses. *C*, phagocytosis of FITC-photoreceptor outer segments (*green*) in WT and PYGM^−/−^ RPE cells stained with TOTO-3 (*magenta*) and Hoechst (*blue*). Scale bar represents 20 μm. *D*, quantitative analysis of phagocytosis using a paired *t* test. Each data point is a biological replicate with two technical replicates each. Error bars show SEM. ∗*p* < 0.05, ∗∗*p* < 0.01, ∗∗∗*p* < 0.001, # *p* < 0.0001. PEDF, pigment epithelium-derived factor; VEGF, vascular endothelial growth factor.

The transepithelial resistance (TER) (28) of freshly picked RPE cells plated on a transwell insert was assessed weekly for 77 days post-plating. *PYGM*^*−/−*^ RPE had significantly lower TER than WT control (Fig. 5*A*). Mutant RPE also secreted increased levels of the cytokines, pigment epithelium-derived factor (PEDF), and vascular endothelial growth factor (VEGF), into the apical and basal medium, respectively (Fig. 5*B*). High VEGF secretion can be indicative of leaky tight junctions (29). However, both mutant and WT RPE showed similar levels of POS phagocytosis (Fig. 5, *C* and *D*), suggesting that this process was not affected by PYGM loss.

### Mutant RPE cells have higher glycogen reserves and a higher basal oxygen consumption rate

**Figure 6:**
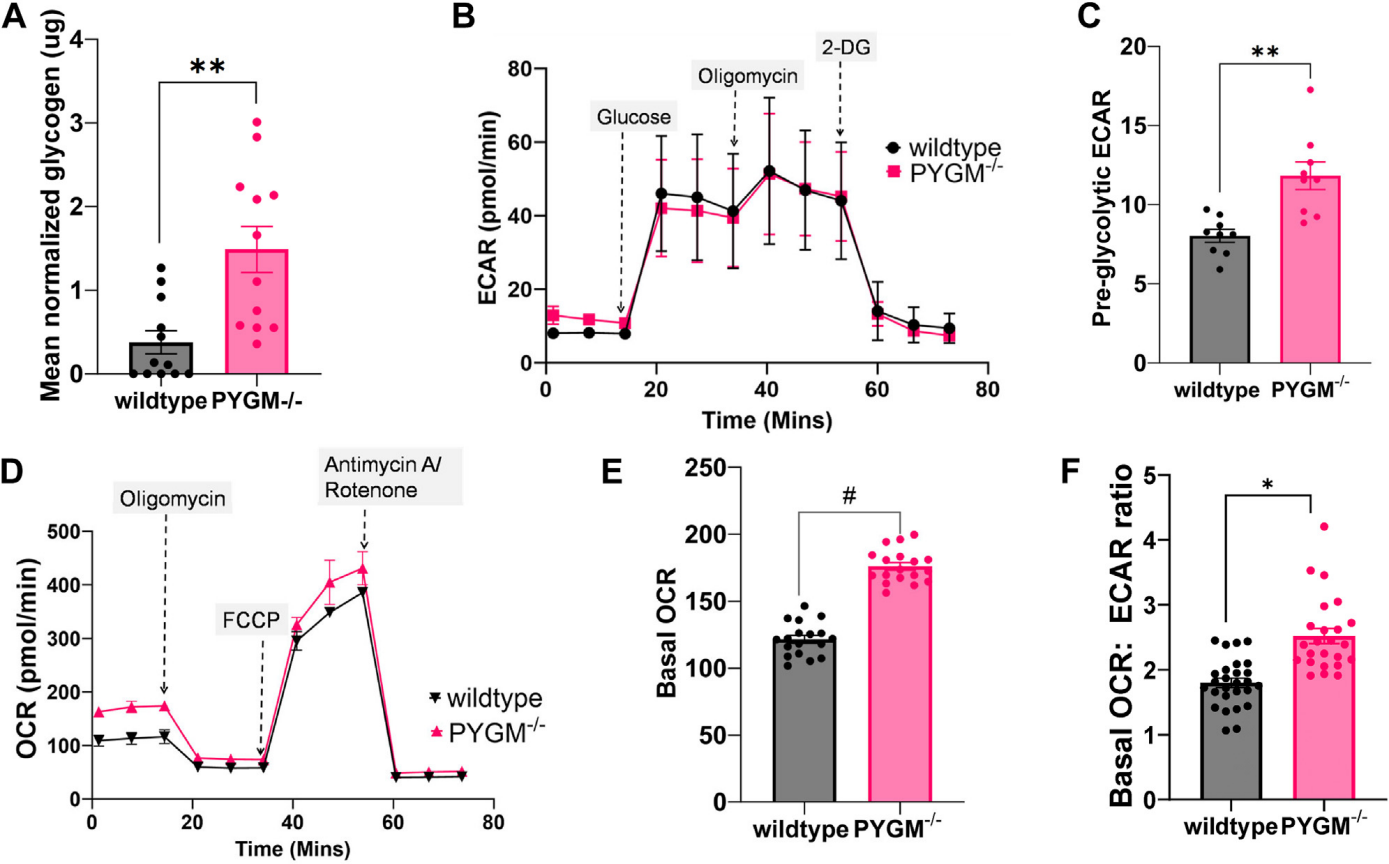
**Metabolic assays comparing WT and *PYGM***^***−/−***^**RPE cells.***A*, glycogen levels were significantly higher in mutant RPE cells. The Mann-Whitney *U*-test was used for analysis since the control data was not normally distributed (as per the Shapiro–Wilk test). Data points represent six technical replicates each of two biological replicates. *B*, glycolytic flux measurement in WT and PYGM^−/−^ RPE using Seahorse. Each point represents three biological replicates with six technical replicates. *C*, pre-glycolytic ECAR in RPE cells analyzed by an unpaired Student’s *t* test. *D*, oxidative phosphorylation measured in WT and *PYGM*^*−/−*^ RPE using Seahorse. Data points represent six technical replicates each of two biological replicates. *E*, basal OCR in in WT and *PYGM*^*−/−*^ RPE analyzed by an unpaired Student’s *t* test. Data was confirmed to be normally distributed by the D’Agostino and Shapiro–Wilk test. Data points represent 3 to 6 technical replicates each of four biological replicates. *F*, basal OCR:ECAR ratio of WT and *PYGM*^*−/−*^ RPE cells. Each data point is a technical replicate of a total of three biological replicates. Data analyzed by unpaired Student’s t-tests. Error bars show SEM. ∗*p* < 0.05, ∗∗*p* < 0.01, ∗∗∗*p* < 0.001, # *p* < 0.0001.

Mutant RPE cells contained higher levels of glycogen (Fig. 6*A*). This was expected because PYGM is a glycogen breakdown enzyme and glycogen reserves would likely increase in the presence of a nonfunctional mutant. The glycolytic profiles of mutant and WT cells, analyzed using an Agilent Seahorse XF instrument to assay real-time metabolic changes, revealed no significant differences in glycolysis (Fig. 6*B*). However, the mutant RPE showed a higher pre-glycolytic extracellular acidification rate (ECAR) (Fig. 6, *B* and *C*), suggesting an increased glycolytic flux due to either lactate produced by anaerobic glycolysis or CO_2_ produced in the TCA cycle. The oxidative phosphorylation profile of these cells, measured using a Seahorse Mito Stress Test to assess mitochondrial function, showed that the mutant and WT cells had similar profiles (Fig. 6*D*). However, mutant cells had a higher basal metabolic activity as demonstrated by the basal oxygen consumption rate (OCR) and a higher OCR:ECAR ratio (Fig. 6, *E* and *F*).

### Mutant RPE cells display higher dependence on glucose in the presence of lactate

**Figure 7:**
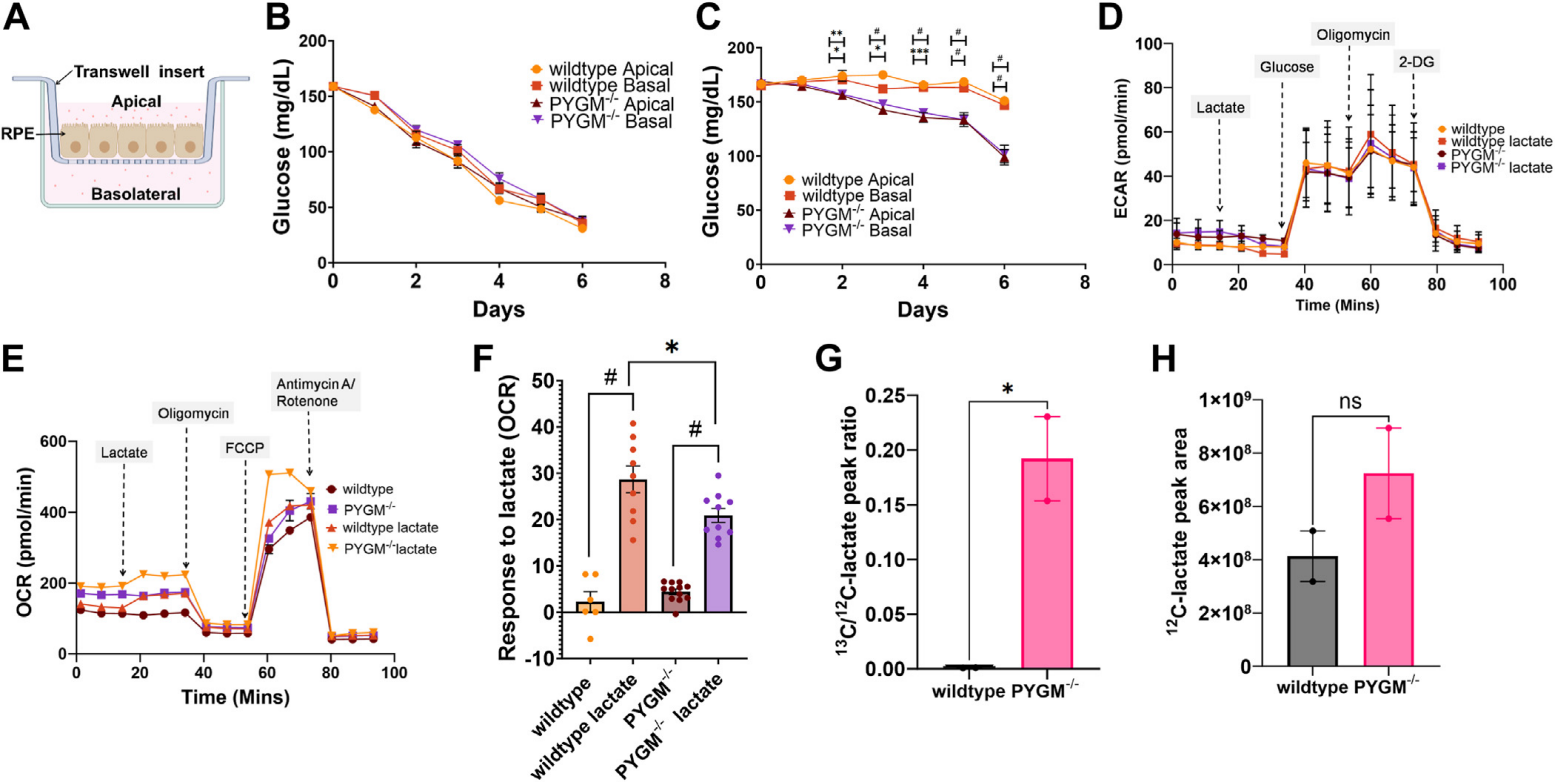
**Metabolic assays comparing WT and *PYGM***^***−/−***^**mutant RPE cells.***A*, schematic of RPE cells growing on a transwell insert showing the apical and basal compartments containing media. *B*, daily glucose levels in apical and basal media from WT and PYGM^−/−^ cells cultured on inserts. Each data point represents four biological replicates. *C*, daily glucose levels in apical and basal media from WT and *PYGM*^*−/−*^ cells apically supplemented with 10 mM sodium lactate. Each point represents two biological replicates. *D*, ECAR profiles of WT and mutant RPE with and without lactate. Each point represents three biological replicates and six technical replicates of each. *E*, OCR profiles of WT and mutant RPE with and without lactate. Each point represents two biological replicates and six technical replicates of each. *F*, acute OCR response on 10 mM lactate addition. The data points represent technical replicates from two biological replicates with three to six technical replicates each. Data analyzed with one-way ANOVA followed by Tukey’s multiple comparison test. *G*, ratio of ^13^C- to ^12^C-lactate in the apical medium 48 h after apical addition of 10 mM ^13^C-lactate in WT and mutant cells, analyzed by unpaired Student’s *t* test. Data points represent two biological replicates. *H*, ^12^C-lactate in the apical medium 48 h after addition of 10 mM ^13^C-lactate apically in WT and PYGM^−/−^ cells, analyzed by unpaired Student’s *t* test. Data points represent two biological replicates. Error bars show SEM. ∗*p* < 0.05, ∗∗*p* < 0.01, ∗∗∗*p* < 0.001, # *p* < 0.0001.

We examined the glucose utilization profile of polarized RPE cultured in inserts (Fig. 7*A*) over a period of 6 days and found that glucose was depleted similarly from both WT and mutant culture media (Fig. 7*B*). This was unexpected, since we reasoned that glucose would deplete more rapidly in the mutant because it would not be able to breakdown glycogen reserves to support metabolic demands. Since normal, healthy RPE cells preferentially use lactate released by photoreceptors (19) in order to spare glucose for photoreceptor use, we wanted to see if mutant RPE could support this function. We therefore assayed glucose utilization by WT and mutant RPE in the presence of 10 mM apical lactate. Although both WT and mutant RPE were able to utilize lactate and spare glucose in the medium, mutant RPE cells were significantly impaired in mediating this metabolic process. Consequently, significantly lower levels of glucose were retained in the culture media of mutant cells (Fig. 7*C*). We supplemented Seahorse assays of glycolysis and oxidative phosphorylation with lactate (Fig. 7, *D* and *E*) and found that WT cells utilized lactate better, as demonstrated by a significantly higher OCR (along with a concomitant decrease in ECAR (Fig. 7*D*)), on lactate addition, than *PYGM*^*−/−*^ cells (Fig. 7, *E* and *F*). To confirm this, we supplemented the apical medium with 10 mM ^13^C-labeled lactate for WT and mutant cells and then assessed the levels of ^12^C- and ^13^C-lactate in the apical medium 48 h after addition using liquid chromatography-mass spectrophotometry. We chose this timepoint because this was the earliest time that we observed significant differences in apical glucose levels after addition of lactate (Fig. 7*C*). The liquid chromatography-mass spectrophotometry data showed that the WT RPE had completely utilized ^13^C-lactate by 48 h and it was no longer detectable in the apical medium. However, significant levels of ^13^C-lactate were still present in mutant RPE culture medium (Fig. 7*G*). As a control, we examined levels of ^12^C-lactate, and these were present in both RPE samples with no significant differences (Fig. 7*H*).

## Discussion

PYGM loss causes McArdle disease or glycogen storage disease (type V) and has been associated with vision loss due to atrophy of the outer retina and RPE, manifesting as a pattern retinal dystrophy. This work reports the derivation and analysis of an iPSC-based model to examine the effects of PYGM loss on RPE cells *in vitro*. The *PYGM* ^−/−^ iPSC line has biallelic frameshift mutations in the first exon of *PYGM*, both predicted to cause NMD. The most common mutation in McArdle disease is a conversion of an arginine at position 50 to a termination codon (p.R50∗) (2, 30), known to undergo NMD (22, 31). Molecular characterization therefore confirms that our null mutant iPSC line is a model that reiterates disease phenotypes due to PYGM loss. Previous animal models include *Pygm* mouse lines carrying the common mutation p.50∗ (32, 33) and a herd of Merino sheep that is PYGM-deficient (34). These models have been studied extensively for skeletal muscle abnormalities which reiterate human disease but, to our knowledge, have not been examined for retinal phenotypes. Our studies of the iPSC line therefore comprise the first molecular investigation of potential retinal pathomechanisms in McArdle maculopathy.

As expected, *PYGM* mutant RPE cells contain higher levels of glycogen since they are unable to break down their stores using PYGM. Breakdown of intracellular glycogen to glucose in RPE cells is important because it buffers and supplements the glucose that is delivered from the choroid to the photoreceptors by the RPE (18). The most obvious pathomechanism that explains why deficiency of a glycogen phosphorylase isoenzyme causes RPE atrophy is that the cells are starved of glucose leading to degeneration. In addition, glycogen accumulation is thought to be a hallmark of senescence in various cell types, and breakdown of glycogen to glucose by phosphorylases prevents premature senescence (35). High glycogen levels can also lead to elevated ROS levels that cause cellular damage (35). In fact, blocking glycogen phosphorylase in cancer cells to raise ROS levels has been proposed as a potential therapeutic strategy (36). Seahorse metabolic stress tests showed that WT and *PYGM* mutant RPE had a similar glycolytic profile, except that the mutant cells showed a higher preglycolytic ECAR. Although glycolytic acidification is mainly due to the conversion of pyruvate to lactate and H^+^, preglycolytic ECAR (prior to adding glucose) is usually due to CO_2_ formation by the TCA cycle or the breakdown of glycogen by a phosphorylase such as PYGM. Since the mutant lacks this enzyme, the elevated preglycolytic ECAR was presumably due to higher TCA cycle activity (37). On examination, the mutant cells did indeed show higher TCA cycle activity as seen by the higher basal OCR than the wildtype. This suggested that the mutant cells have a higher energy requirement.

**Figure 8:**
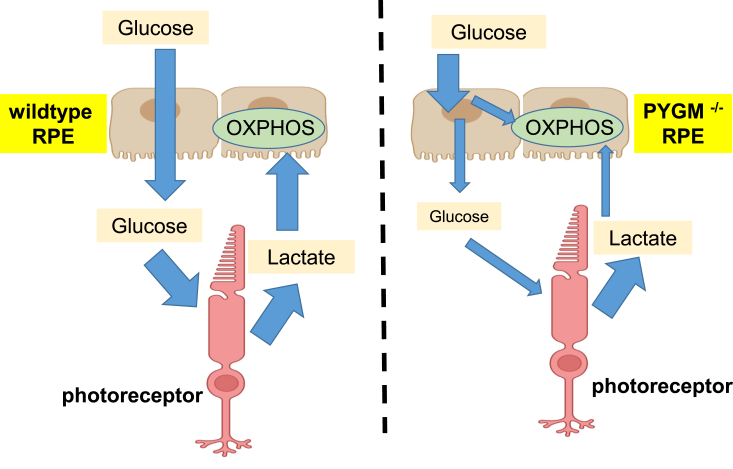
**Schematic showing glucose and lactate utilization by WT and *PYGM***^***−/−***^**RPE cells.** WT RPE pass most of the glucose coming in from the bloodstream to the photoreceptors to use as an energy source for aerobic glycolysis. The photoreceptors in turn release lactate that can be used to fuel oxidative phosphorylation (OXPHOS) in RPE cells thus sparing glucose for photoreceptor use. WT RPE cells use lactate more efficiently than *PYGM*^*−/−*^ RPE and the mutant cells have greater reliance on glucose as a fuel.

Normally, photoreceptor cells use glucose for aerobic glycolysis, whereas RPE cells primarily use oxidative phosphorylation to meet their energy requirements (19). Glucose comes into RPE cells from the choroid bloodstream and any surplus is transferred from RPE to photoreceptor cells where it is used to power aerobic glycolysis that generates lactate as one of the end-products. Released lactate is taken up by RPE cells and converted to pyruvate, which is fed into oxidative phosphorylation to fuel RPE cells. In fact, RPE cells preferentially use lactate to spare glucose for photoreceptor use (19). Daily measurements of apical and basal glucose levels for WT and mutant RPE cultures showed that they used glucose at a similar rate. However, when we supplemented the apical medium with lactate, the WT cells had considerably more glucose left over in the medium. This suggests that in the presence of lactate, the mutant RPE cells use more glucose than WT RPE cells, resulting in less glucose being available to release apically for photoreceptor use (Fig. 8). We therefore suggest that a plausible pathomechanism is suboptimal glucose availability to photoreceptor cells that, over years, is a key contributor to retinal degeneration and loss of vision in individuals with McArdle disease.

Loss of PYGM could affect other RPE functions including cellular metabolism and cell-cell junctions, in addition to the direct consequences of defective glycogen breakdown. RPE cells regenerate 11-*cis* retinal from all-*trans* retinal to make it available for photoreceptor function during the visual cycle. The final step occurs in RPE cells when 11-*cis* retinol is oxidized to 11-*cis*-retinal by the retinol dehydrogenases RDH5 and RDH11 (38). Loss of PYGM resulted in significant downregulation of *RDH11* expression in RPE cells, which may be a synergistic effect because retinoic acid metabolism and energy metabolism are closely interconnected. For example, fasting or diabetes affects expression of genes involved in retinoic acid metabolism in the liver, with levels of RDH11 significantly downregulated (39). Normal RPE cells also display polarized secretion of cytokines VEGF and PEDF. VEGF is secreted basally to induce vascularization (40, 41), whereas PEDF is secreted apically where it has a neuroprotective role in the retina (42, 43). In chick, PEDF is reported to activate cone-specific gene expression and decrease the number of rod cells (44). Elevated levels of these cytokines, observed in *PYGM*^*−/−*^ mutant RPE, suggest defects in polarization. Additionally, high PEDF secretion is reported in RPE from AMD patients (45) and may have functional implications for rod survival. VEGF is also important for the survival of Müller cells and photoreceptors, in addition to its role in vasculogenesis (46). Therefore, even though individuals with McArdle disease do not show neovascularization of the retina (47), elevated secretion of VEGF may still affect retinal function. We observed that mutant RPE cells had lower TER, which is linked to increased tight junction permeability (48) and at the ultrastructural level, has been correlated with a decrease in tight junction membrane contact points (49). This, in addition to higher levels of secreted cytokines in the apical and basal media collectively, suggests leaky cell–cell junctions (29, 48, 49). This is likely to impair the ability of these cells to polarize and form a tight barrier that is essential for optimal RPE function.

The virtue of the model system we have used is its simplicity. It allows us to observe the effects of the null mutation on the cellular phenotype and function of RPE cells and to identify key biochemical defects. However, this model does not fully mimic all of the *in vivo* RPE cell states and interactions. For instance, McArdle maculopathy is not generally observed in young patients because it is a disease of aging retina (6, 47). Furthermore, RPE cells in the retina phagocytose POSs on a daily basis. Some of these segments carry *bis*-retinoids, which over time lead to the build-up of lipofuscin deposits that are associated with RPE cell death (50, 51). The phagocytosis of POSs also fuels beta-oxidation of fatty acids in RPE (52), which may then indirectly affect glucose utilization by these cells. We have also not examined the effects of the *PYGM* null mutation, if any, on the photoreceptor cells which are known to express PYGM in mice (53).

In summary, our data suggest that these defects in *PYGM*-mutated individuals are a combination of glucose stress, leaky tight junctions, and the burden associated with high glycogen reserves. It would be of benefit to investigate if dietary intake of alternative substrates that are used by RPE cells to generate TCA cycle intermediates, for example, proline (54, 55), could reduce the glucose dependency displayed by *PYGM*^−/−^cells. Since this is a null mutation, gene augmentation could be a viable therapeutic intervention for later onset vision impairment associated with McArdle disease. The iPSC-derived model that we have developed for studying vision defects associated with PYGM loss will be useful for studying other cellular and biochemical aspects of this disease, as well as in screens for the drugs or metabolites aimed at developing new therapeutic interventions.

## Experimental procedures

### iPSC culture

Human iPSCs were cultured on six-well plates on Matrigel GFR (Corning, 354230)-coated wells with mTeSR Plus (Stemcell Technologies, 100-0276) media. Cell culture medium was replaced on a daily basis with double feed on a weekend (skipped 1 day a week). Cells were allowed to grow for 4 to 5 days prior to passaging or induction of differentiation. Passaging was carried out using ReleSR (StemcellTech, 100-0484) solution at 37 °C for 5 to 7 min, and cells were transferred to fresh matrigel plates in a 1:20–1:30 ratio. All cultures were maintained at 37 °C, in a humidified environment, with 5% CO2. Cells were cryopreserved with freezing media containing 90% fetal bovine serum (Gibco, 10270), 10% dimethyl sulfoxide (Sigma, D2650), and 10 μM Y-27632 (Chemdea, CD0141). *Mycoplasma* tests were carried out at regular intervals. The cell line AD2 was a generous gift from Prof. Majlinda Lako, Newcastle University and it has previously been referenced as WT1 (23). The authenticity and genomic status of this iPSC line has been validated by array CGH and karyotyping, as described previously (23).

### Introducing PYGM mutation in iPS cells

Benchling was used to design guides that would efficiently target exon 1 of *PYGM*. The guide sequence chosen was 5′- ACG AGA CTA CTA CTT TGC TC as it was closest to the p.R50∗ McArdle mutation. Equimolar quantities of cRNA with the guide sequence and the fluorescently-labeled ATTO-550 tracrRNA (Integrated DNA Technologies (IDT), 1075927) were incubated as per manufacturer’s instructions to obtain a functional guide RNA duplex. The Cas9-GFP containing plasmid, pSpCas9-(BB)-2A-GFP (Addgene, 48138) was cotransfected with the tracrRNA:crRNA mixture using Lipofectamine Stem transfection reagent (Thermo Fisher Scientific, STEM00003). After 48 h, the transfected WT cells were sorted into single cells based on Cas9-GFP expression using FACS (BD Influx 6 way cell sorter). The sorting buffer and the culture medium were supplemented with 10 μM Y-27632 (Chemdea, CD0141). Resulting colonies were split and genotyped after 10 days. TIDE analysis (https://tide.nki.nl/) and ICE analysis Synthego (https://ice.synthego.com) were used to identify mutants. Mutation taster (26) and NMD escape predictor (25) were used to predict if these mutants were likely to be null. The lines were sequenced to rule out any off-target mutations using the listed primers (Table S2). The mutant line and the control line were confirmed to be isogenic.

### Antibody staining of iPS cells

iPS cells were plated on matrigel-coated coverslips and after 48 h, they were fixed with 4% paraformaldehyde for 15 min before staining for antibodies. The Oct3/4 antibody (AF1759, R&D Systems, Inc) was used at 10 μg/ml and the preconjugated SSEA4 Alexa Fluor 555 antibody (560218, BD Bioscience) at 1:50. After incubation with primary antibody for 90 min, the cells were washed and incubated with anti-goat IgG Alexa Fluor 488 conjugated secondary antibody at 1:2000 for 90 min. The cells were washed and stained with DAPI before mounting on slides with Prolong Gold (Molecular Probes). Antibody specificities were tested by the manufacturers. Details of all antibodies used are available in Table S3.

### Differentiating iPS cells to RPE cells

iPSC colonies were grown to 80 to 95% confluency in 6-well plates. The iPSC culture medium was replaced with 2 ml of differentiation medium [Advanced RPMI 1640, (12633, Gibco), GlutaMAX-1 (35050, Gibco), penicillin/streptomycin (Gibco, 15140), and B-27 (Gibco, 17504)] supplemented with 10 μM SB431542 (STEMCELL, 72232) and 10 ng/μl Noggin (R&D Systems, 6057-NG-025) from days 0 to 5. From days 6 to 9, only 10 ng/μl Noggin (R&D Systems, 6057-NG-025) was added to the medium. From days 10 to 15, the medium was supplemented with 5 ng/μl activin A (PeproTech, 120–14 A), and from days 16 to 21, activin A was replaced with 3 μM CHIR99021 (Sigma, SML1046). The cells were then fed every 2 days until the first RPE patches appeared, normally by week 8 of differentiation. RPE patches were mechanically picked and placed in TryPLE Select (10×) (Invitrogen) for a maximum of 30 min to dissociate the cells. Cells were sieved using a 70 μm cell strainer and replated at 4.5 × 10^5^ cells per cm^2^ on 24-well plates or on 0.33 cm^2^ PET hanging cell culture inserts (Merck Millipore) coated with matrigel.

### Measurement of trans-epithelial resistance

TER was performed using a Millicell ERS-2 Voltohmmeter (Millipore, MERS00002) by measuring the resistance of the blank transwell insert with culture medium as a control reading and then measuring the inserts with RPE cells. The shorter and longer tips of the electrode were inserted in the transwell apical chamber and in the basolateral chamber, respectively. The resistance was measured twice in each transwell insert. The resistance reading of the blank was then subtracted from the resistance reading of the cells for each measurement. The results were multiplied by the membrane area value using the formula: Unit area resistance = Resistance (Ω) × effective membrane area (cm^2^), where the final value was given in ohms (Ω).

### Phagocytosis assay

RPE cells were plated on “Cell Carrier Ultra” Operetta plates 4 to 5 weeks prior to the experiment. Bovine rod POS were isolated from bovine retina using a previously published protocol (56) and frozen as aliquots of 1X10^6^ POS. On the day of the experiment, an appropriate number of frozen POS aliquots were thawed and centrifuged at 2600*g* for 4 min, and the pellet was resuspended in 1 ml medium (AdRPMI 1640 (12633, Gibco) + B-27 Supplement (Gibco, 17504) + 10% heat-inactivated fetal bovine serum (Gibco, 10270)). Following the addition of 0.4 mg/ml FITC (Sigma, F7250), the POS were incubated for 1 h at room temperature while agitating in the dark. POS were centrifuged at 2600*g* for 4 min again and washed three times with medium and the staining was confirmed under a Bioscience Axiovert microscope. RPE cells were treated with 1 × 10^6^ POS-FITC per cm^2^ and incubated for 4 h at 37 °C. For the control experiments, RPE cells were treated with the same number of nonstained POS and incubated for the same time. The cells were then washed twice with fresh medium and stained with Deep Red cell mask (Thermo Fisher Scientific, C10046), (1:1000) and Hoechst (Thermo Fisher Scientific, H1399), (1:2000). We added 0.2% Trypan Blue (Sigma, T8154) in PBS for 5 min to the test wells for quenching the FITC on the external, bound POS. The plate was imaged on an Operetta high content microscope and analyzed using the Columbus software, PerkinElmer.

### RPE cytokine secretion studies

Medium from basal and apical chambers of transwell inserts were collected from WT and mutant RPE cells. The levels of PEDF and VEGF secretion were measured by using human PEDF-ELISA (Human Serpin F1/PEDF DuoSet, 5 Plate, DY008, R&D Technologies)and human VEGF-ELISA Kit (Human VEGF DuoSet ELISA, 31330095, R&D Technologies) along with the DuoSet Ancillary Reagent Kit (CF962, R&D Technologies) according to the manufacturer’s instructions. The plate was read on a Berthold plate reader.

### Metabolic analysis

Real-time ECAR and OCR of WT and mutant RPE cells were analyzed with a XF-96 Extracellular Flux Analyzer (Seahorse Bioscience) using either the Glycolysis Stress Kit (Agilent, 103020-100) or the Mito Stress Kit (Agilent, 013015-100). Cells were plated (six wells per treatment) on Seahorse plates coated with matrigel and allowed to mature for 8 weeks before the experiment. Basal metabolic rates were determined in unbuffered Seahorse base medium (Agilent, 103334-100) containing 1 mM L-glutamine for the glycolysis stress test and containing 1 mM pyruvate, 2 mM L-glutamine, and 10 mM glucose for the Mito Stress Test measurements. For both media, the pH was adjusted to 7.4 with 0.1 M NaOH prior to use. For assessing glycolysis, three basal measurements were taken and then glucose (10 mM), oligomycin (1 μM), and 2-DG (50 mM) were added sequentially, and three measurements were made after each addition according to the manufacturer’s protocol. Similarly, for assessing oxidative phosphorylation, three basal measurements were taken and then oligomycin (2 μM), FCCP (2 μM), and antimycin/rotenone (0.5 μM) were added sequentially and three measurements were made after each addition. For testing the effects of lactate, sodium lactate was added following three initial basal measurements, to a final concentration of 10 mM, followed by three measurements before adding the other drugs. Flux measurements were normalized to cellular DNA and protein using a Crystal Violet assay and read on a Berthold plate reader.

### Glycogen content measurement

Glycogen measurements of cells were done using a kit (AbCam, ab169558) according to the manufacturer’s instructions. RPE cells grown on inserts and allowed to mature were dissociated using 10× TrypLE solution and counted. They were then spun down and resuspended in double distilled water and boiled for 10 min to release the glycogen into the solution. The debris was then spun down and the supernatant used in the glycogen assay. The experiments used 57,500 cells/well of WT and mutant.

### Measurement of glucose levels in media

Glucose levels were measured in media using the GlucCell meter (KDBio) using the manufacturer’s instructions. Briefly, 2.5 μl of medium was added to the measurement strip and the glucose concentration read on the machine. This was done for both apical and basal samples. For media supplementation, sodium lactate (Fisher chemicals, 11334278) was added to a final concentration of 10 mM. Media pH was unaffected after lactate addition.

### Mass spectrometric analysis of media

Media from mutant and WT samples were collected 48 h after lactate addition. Each sample was analyzed twice-once in positive and once in negative ionization mode. For each run, 1 μl sample in 5% methanol was injected on to a Vanquish LC system (Thermo Fisher Scientific) using a flow rate of 0.3 ml/min. Sample was separated using a Hypersil GOLD C18 column (1.9 μm particle size, 150 mm × 2.1 mm) held at 30 °C. Starting mobile phase composition was 1% solvent B (0.1% formic acid in acetonitrile) in A (0.1% formic acid in water) for 1 min followed by a gradient elution of 1% B to 95% B in 6 min. The column was washed at 95% B for 4 min followed by re-equilibration at 1% B for 4 min. Separated compounds were eluted in to an Orbitrap Exploris 240 mass spectrometer and ionized using electrospray ionization. Capillary voltage was 3.5 kV for positive mode and 2.5 kV for negative mode. Mass measurement used full scan mode resolution of 120,000, at an m/z range of 80 to 800. The maximum injection time was set automatically by the software. Individual samples were analyzed in MS only mode. For compound identification, a system suitability testing sample was prepared by mixing 3 μl of each sample, this sample was analyzed three times using an AcquireX deep scan acquisition workflow, which injects the sample three times and dynamically updates inclusion and exclusion lists after each injection to maximize the number of identified compounds. A blank injection was used to establish background signals which were added to an exclusion list before system suitability testing analysis. MS settings were as above. Intensity threshold for MS/MS selection was 5000; dynamic exclusion was set to 10 s after one time. Fragmentation in a higher-energy collisional dissociation cell used a stepped relative collision energy of 30, 50, and 150% and measured with a resolution of 15,000. Raw data was processing using Compound Discoverer 3.1 for retention time alignment, feature annotation, QC correction, and relative quantitation of labeled *versus* unlabeled metabolites. Details of Compound Discoverer settings are provided in Table S4.

### RNA extraction and reverse transcriptase PCRs

RNA was extracted from cell pellets using Trizol (Ambion) according to the manufacturer’s protocol. The RNA was treated with DNaseI (Ambion) to get rid of any contaminating DNA. The RNA was then purified using RNA clean and concentrator columns (Zymo research). One microgram of pure RNA was taken forward to generate complementary DNA using the Superscript III reverse transcriptase using the manufacturer’s instructions. The complementary DNA was taken forward for PCRs using the Hotshot Diamond 2× PCR mix and specific primers (Table S1). The RT-PCRs were repeated a minimum of 2 to 3 times and the gels were imaged used Bio-Rad chemidoc, ensuring no saturation. The bands were subsequently analyzed using ImageLab software (https://www.bio-rad.com/en-uk/product/image-lab-software) and normalized using the GAPDH band, which served as a loading control.

### Immunoblotting

Whole cell extracts were prepared from mature RPE samples using protein extraction buffer (Pierce) and resolved using NuPAGE Bis-Tris gradient gel using MES running buffer as per the manufacturer’s protocol. The gels were transferred onto Immuno-FL membranes. Primary antibodies PYGM (1:500), RDH11 (1:1000), and ACTB (1:10,000) were used overnight in blocking buffer. Li-Cor secondary antibodies IRDye680 and IRDye800against rabbit and mouse were used at 1:10,000 on the blot and imaged on a BioRad imaging system for multichannel imaging of test and control antibody simultaneously. Image lab was used to quantify bands. Uncropped, original immunoblots are shown in Fig. S2, *A* and *B*.

### RPE characterization by immunocytochemistry

Cells were fixed in 4% formaldehyde (Sigma, 47,608) for 15 min at room temperature and permeabilized with 0.25% Triton X-100 (Sigma, T8787) for 15 min, followed by treatment with blocking solution (3% normal goat serum in PBS+0.02% Triton-X-100, CHX-9067, Chondrex) for 30 min at room temperature. Cells were treated with the following primary antibodies anti-bestrophin (Abcam, ab2182, 1:300), anti-sodium potassium ATPase (Alexa Fluor 488 conjugate) (Abcam, ab197713, 1:50), MERTK (Bethyl, A300-222A, 1:200), collagen IV (Abcam, ab6586, 1:200) overnight at 4 °C and then with secondary antibodies anti-rabbit 488 or anti-mouse 568/647 (Thermo Fisher Scientific, 1:2000) diluted in blocking solution for 90 min at room temperature. Washes with 1XPBS were carried out between and after treatments. Prior to mounting, cells were stained with DAPI (Molecular Probes) and imaged using a Nikon A1R confocal microscope in combination with the associated NIS Elements software. All antibody details are shown in Table S3.

### Statistical analysis

*p* values of normally distributed data sets were calculated using two-tailed Student’s *t* test, or one-way ANOVA with Tukey’s *post hoc* test, or two-way ANOVA with Bonferroni *post hoc* tests using GraphPad Prism Software Inc. Normality was checked using D’Agostino and Wilk–Shapiro tests. For Seahorse glycolysis stress experiments, the raw data from three experiments were pretreated with a scaling factor for the “baseline readings” prior to collation to ensure that the SDs were representative of experimental differences and not technical variations as outlined for stress tests (57). Error bars represent the S.E.M unless otherwise indicated. The statistical significance of pairwise comparisons shown on bar graphs is indicated by n.s. not significant, ∗*p* < 0.05, ∗∗*p* < 0.01, ∗∗∗*p* < 0.001 and #*p* < 0.0001.

## Data availability

All the data supporting our findings are contained within the manuscript.

## Supporting information

This article contains supporting information.

## Conflict of interest

The authors declare that they have no conflicts of interest with the contents of this article.

## References

[1] Bartram C., Edwards R.H., Beynon R.J. (1995). McArdle's disease-muscle glycogen phosphorylase deficiency. Biochim. Biophys. Acta.

[2] Santalla A., Nogales-Gadea G., Encinar A.B., Vieitez I., Gonzalez-Quintana A., Serrano-Lorenzo P. (2017). Genotypic and phenotypic features of all Spanish patients with McArdle disease: a 2016 update. BMC Genomics.

[3] Bruno C., Cassandrini D., Martinuzzi A., Toscano A., Moggio M., Morandi L. (2006). McArdle disease: the mutation spectrum of PYGM in a large Italian cohort. Hum. Mutat..

[4] Llavero F., Arrazola Sastre A., Luque Montoro M., Galvez P., Lacerda H.M., Parada L.A. (2019). McArdle disease: new insights into its underlying molecular mechanisms. Int. J. Mol. Sci..

[5] McArdle B. (1951). Myopathy due to a defect in muscle glycogen breakdown. Clin. Sci..

[6] Mahroo O.A., Khan K.N., Wright G., Ockrim Z., Scalco R.S., Robson A.G. (2019). Retinopathy associated with biallelic mutations in PYGM (McArdle disease). Ophthalmology.

[7] Shalaby A.K., Charbel Issa P. (2021). Retinopathy in McArdle disease. Ophthalmol. Retina.

[8] Alsberge J.B., Chen J.J., Zaidi A.A., Fu A.D. (2021). RETINAL DYSTROPHY IN A PATIENT WITH McARDLE DISEASE. Retin. Cases Brief Rep..

[9] Vaclavik V., Naderi F., Schaller A., Escher P. (2020). Longitudinal case study and phenotypic multimodal characterization of McArdle disease-linked retinopathy: insight into pathomechanisms. Ophthalmic Genet..

[10] Leonardy N.J., Harbin R.L., Sternberg P. (1988). Pattern dystrophy of the retinal pigment epithelium in a patient with McArdle's disease. Am. J. Ophthalmol..

[11] Migocka-Patrzalek M., Elias M. (2021). Muscle glycogen phosphorylase and its functional partners in health and disease. Cells.

[12] Zhao C.Y., Hua C.H., Li C.H., Zheng R.Z., Li X.Y. (2021). High PYGL expression predicts poor prognosis in human gliomas. Front. Neurol..

[13] Hernandez C., Garcia-Ramirez M., Garcia-Rocha M., Saez-Lopez C., Valverde A.M., Guinovart J.J. (2014). Glycogen storage in the human retinal pigment epithelium: a comparative study of diabetic and non-diabetic donors. Acta Diabetol..

[14] Kurihara T., Westenskow P.D., Gantner M.L., Usui Y., Schultz A., Bravo S. (2016). Hypoxia-induced metabolic stress in retinal pigment epithelial cells is sufficient to induce photoreceptor degeneration. Elife.

[15] Louer E.M.M., Yi G., Carmone C., Robben J., Stunnenberg H.G., den Hollander A.I. (2020). Genes involved in energy metabolism are differentially expressed during the day-night cycle in murine retinal pigment epithelium. Invest. Ophthalmol. Vis. Sci..

[16] Strauss O. (2005). The retinal pigment epithelium in visual function. Physiol. Rev..

[17] Yang S., Zhou J., Li D. (2021). Functions and diseases of the retinal pigment epithelium. Front. Pharmacol..

[18] Senanayake P., Calabro A., Hu J.G., Bonilha V.L., Darr A., Bok D. (2006). Glucose utilization by the retinal pigment epithelium: evidence for rapid uptake and storage in glycogen, followed by glycogen utilization. Exp. Eye Res..

[19] Kanow M.A., Giarmarco M.M., Jankowski C.S., Tsantilas K., Engel A.L., Du J. (2017). Biochemical adaptations of the retina and retinal pigment epithelium support a metabolic ecosystem in the vertebrate eye. Elife.

[20] Viegas F.O., Neuhauss S.C.F. (2021). A metabolic landscape for maintaining retina integrity and function. Front. Mol. Neurosci..

[21] Tarraso G., Real-Martinez A., Pares M., Romero-Cortadellas L., Puigros L., Moya L. (2020). Absence of p.R50X Pygm read-through in McArdle disease cellular models. Dis. Model. Mech..

[22] Sohn E.H., Kim H.S., Lee A.Y., Fukuda T., Sugie H., Kim D.S. (2008). A novel PYGM mutation in a Korean patient with McArdle disease: the role of nonsense-mediated mRNA decay. Neuromuscul. Disord..

[23] Buskin A., Zhu L., Chichagova V., Basu B., Mozaffari-Jovin S., Dolan D. (2018). Disrupted alternative splicing for genes implicated in splicing and ciliogenesis causes PRPF31 retinitis pigmentosa. Nat. Commun..

[24] Sanjurjo-Soriano C., Erkilic N., Baux D., Mamaeva D., Hamel C.P., Meunier I. (2020). Genome editing in patient iPSCs corrects the most prevalent USH2A mutations and reveals intriguing mutant mRNA expression profiles. Mol. Ther. Methods Clin. Dev..

[25] Coban-Akdemir Z., White J.J., Song X., Jhangiani S.N., Fatih J.M., Gambin T. (2018). Identifying genes whose mutant transcripts cause dominant disease traits by potential gain-of-function alleles. Am. J. Hum. Genet..

[26] Schwarz J.M., Cooper D.N., Schuelke M., Seelow D. (2014). MutationTaster2: mutation prediction for the deep-sequencing age. Nat. Methods.

[27] May-Simera H.L., Wan Q., Jha B.S., Hartford J., Khristov V., Dejene R. (2018). Primary cilium-mediated retinal pigment epithelium maturation is disrupted in ciliopathy patient cells. Cell Rep..

[28] Wegener J., Abrams D., Willenbrink W., Galla H.J., Janshoff A. (2004). Automated multi-well device to measure transepithelial electrical resistances under physiological conditions. Biotechniques.

[29] Farjood F., Vargis E. (2017). Physical disruption of cell-cell contact induces VEGF expression in RPE cells. Mol. Vis..

[30] Lucia A., Ruiz J.R., Santalla A., Nogales-Gadea G., Rubio J.C., Garcia-Consuegra I. (2012). Genotypic and phenotypic features of McArdle disease: insights from the Spanish national registry. J. Neurol. Neurosurg. Psychiatry.

[31] Nogales-Gadea G., Rubio J.C., Fernandez-Cadenas I., Garcia-Consuegra I., Lucia A., Cabello A. (2008). Expression of the muscle glycogen phosphorylase gene in patients with McArdle disease: the role of nonsense-mediated mRNA decay. Hum. Mutat..

[32] Nogales-Gadea G., Pinos T., Lucia A., Arenas J., Camara Y., Brull A. (2012). Knock-in mice for the R50X mutation in the PYGM gene present with McArdle disease. Brain.

[33] Fiuza-Luces C., Santos-Lozano A., Llavero F., Campo R., Nogales-Gadea G., Diez-Bermejo J. (2018). Muscle molecular adaptations to endurance exercise training are conditioned by glycogen availability: a proteomics-based analysis in the McArdle mouse model. J. Physiol..

[34] Howell J.M., Walker K.R., Creed K.E., Dunton E., Davies L., Quinlivan R. (2014). Phosphorylase re-expression, increase in the force of contraction and decreased fatigue following notexin-induced muscle damage and regeneration in the ovine model of McArdle disease. Neuromuscul. Disord..

[35] Favaro E., Bensaad K., Chong M.G., Tennant D.A., Ferguson D.J., Snell C. (2012). Glucose utilization *via* glycogen phosphorylase sustains proliferation and prevents premature senescence in cancer cells. Cell Metab..

[36] Davidson CD T.J., Amiel E., Carr F.E. (2022). Inhibition of glycogen metabolism induces reactive oxygen species-dependent apoptosis in anaplastic thyroid cancer. bioRxiv.

[37] Pike Winer L.S., Wu M. (2014). Rapid analysis of glycolytic and oxidative substrate flux of cancer cells in a microplate. PLoS One.

[38] Parker R.O., Crouch R.K. (2010). Retinol dehydrogenases (RDHs) in the visual cycle. Exp. Eye Res..

[39] Klyuyeva A.V., Belyaeva O.V., Goggans K.R., Krezel W., Popov K.M., Kedishvili N.Y. (2021). Changes in retinoid metabolism and signaling associated with metabolic remodeling during fasting and in type I diabetes. J. Biol. Chem..

[40] Marneros A.G., Fan J., Yokoyama Y., Gerber H.P., Ferrara N., Crouch R.K. (2005). Vascular endothelial growth factor expression in the retinal pigment epithelium is essential for choriocapillaris development and visual function. Am. J. Pathol..

[41] Saint-Geniez M., Kurihara T., Sekiyama E., Maldonado A.E., D'Amore P.A. (2009). An essential role for RPE-derived soluble VEGF in the maintenance of the choriocapillaris. Proc. Natl. Acad. Sci. U. S. A..

[42] Comitato A., Subramanian P., Turchiano G., Montanari M., Becerra S.P., Marigo V. (2018). Pigment epithelium-derived factor hinders photoreceptor cell death by reducing intracellular calcium in the degenerating retina. Cell Death Dis..

[43] Polato F., Becerra S.P. (2016). Pigment epithelium-derived factor, a protective factor for photoreceptors *in vivo*. Adv. Exp. Med. Biol..

[44] Volpert K.N., Tombran-Tink J., Barnstable C., Layer P.G. (2009). PEDF and GDNF are key regulators of photoreceptor development and retinal neurogenesis in reaggregates from chick embryonic retina. J. Ocul. Biol. Dis. Infor..

[45] An E., Lu X., Flippin J., Devaney J.M., Halligan B., Hoffman E.P. (2006). Secreted proteome profiling in human RPE cell cultures derived from donors with age related macular degeneration and age matched healthy donors. J. Proteome Res..

[46] Saint-Geniez M., Maharaj A.S., Walshe T.E., Tucker B.A., Sekiyama E., Kurihara T. (2008). Endogenous VEGF is required for visual function: evidence for a survival role on muller cells and photoreceptors. PLoS One.

[47] Casalino G., Chan W., McAvoy C., Coppola M., Bandello F., Bird A.C. (2018). Multimodal imaging of posterior ocular involvement in McArdle's disease. Clin. Exp. Optom..

[48] Wang M., Li H., Wang F. (2022). Roles of transepithelial electrical resistance in mechanisms of retinal pigment epithelial barrier and retinal disorders. Discov. Med..

[49] Rajasekaran S.A., Hu J., Gopal J., Gallemore R., Ryazantsev S., Bok D. (2003). Na,K-ATPase inhibition alters tight junction structure and permeability in human retinal pigment epithelial cells. Am. J. Physiol. Cell Physiol..

[50] Sparrow J.R., Boulton M. (2005). RPE lipofuscin and its role in retinal pathobiology. Exp. Eye Res..

[51] Taubitz T., Fang Y., Biesemeier A., Julien-Schraermeyer S., Schraermeyer U. (2019). Age, lipofuscin and melanin oxidation affect fundus near-infrared autofluorescence. EBioMedicine.

[52] Adijanto J., Du J., Moffat C., Seifert E.L., Hurle J.B., Philp N.J. (2014). The retinal pigment epithelium utilizes fatty acids for ketogenesis. J. Biol. Chem..

[53] Corbo J.C., Myers C.A., Lawrence K.A., Jadhav A.P., Cepko C.L. (2007). A typology of photoreceptor gene expression patterns in the mouse. Proc. Natl. Acad. Sci. U. S. A..

[54] Chao J.R., Knight K., Engel A.L., Jankowski C., Wang Y., Manson M.A. (2017). Human retinal pigment epithelial cells prefer proline as a nutrient and transport metabolic intermediates to the retinal side. J. Biol. Chem..

[55] Yam M., Engel A.L., Wang Y., Zhu S., Hauer A., Zhang R. (2019). Proline mediates metabolic communication between retinal pigment epithelial cells and the retina. J. Biol. Chem..

[56] Parinot C., Rieu Q., Chatagnon J., Finnemann S.C., Nandrot E.F. (2014). Large-scale purification of porcine or bovine photoreceptor outer segments for phagocytosis assays on retinal pigment epithelial cells. J. Vis. Exp..

[57] van den Berg R.A., Hoefsloot H.C., Westerhuis J.A., Smilde A.K., van der Werf M.J. (2006). Centering, scaling, and transformations: improving the biological information content of metabolomics data. BMC Genomics.

