# Supplementary figures and images for: Glycogen myophosphorylase loss causes increased dependence on glucose in iPSC-derived retinal pigment epithelium
Source: J Biol Chem. 2024 Jul 14;300(8):107569. doi: 10.1016/j.jbc.2024.107569 (PMC11342771; doi:10.1016/j.jbc.2024.107569)

**A**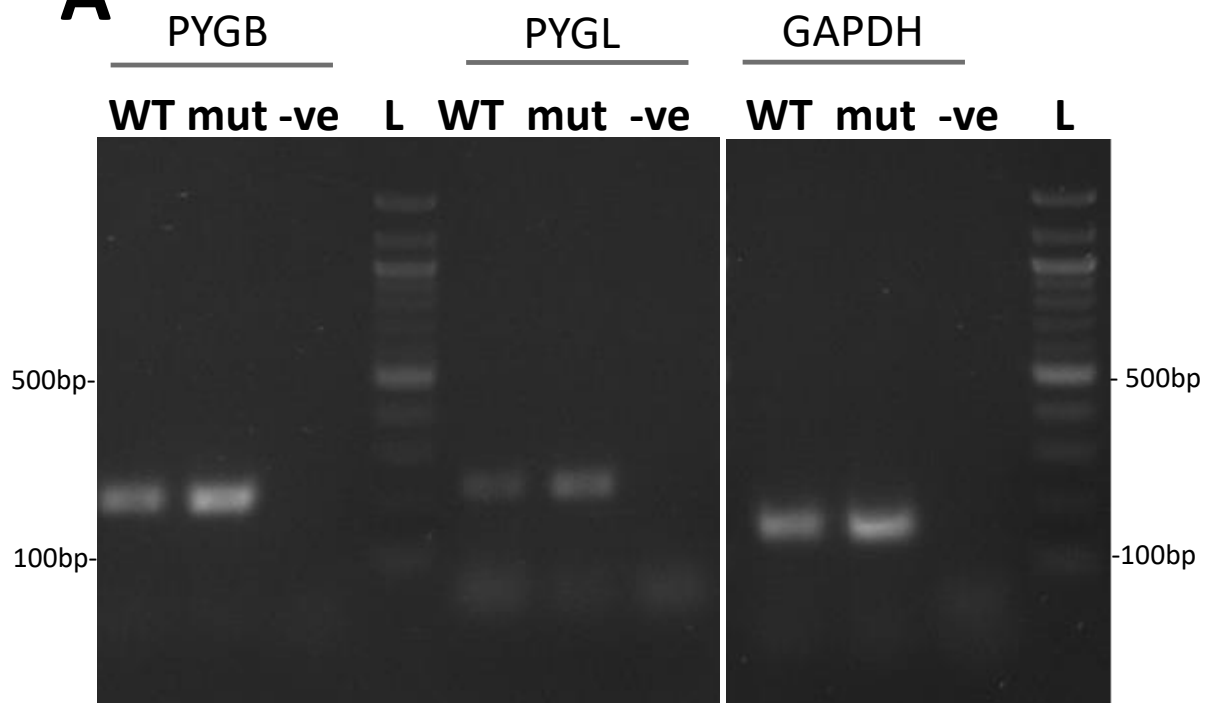**B**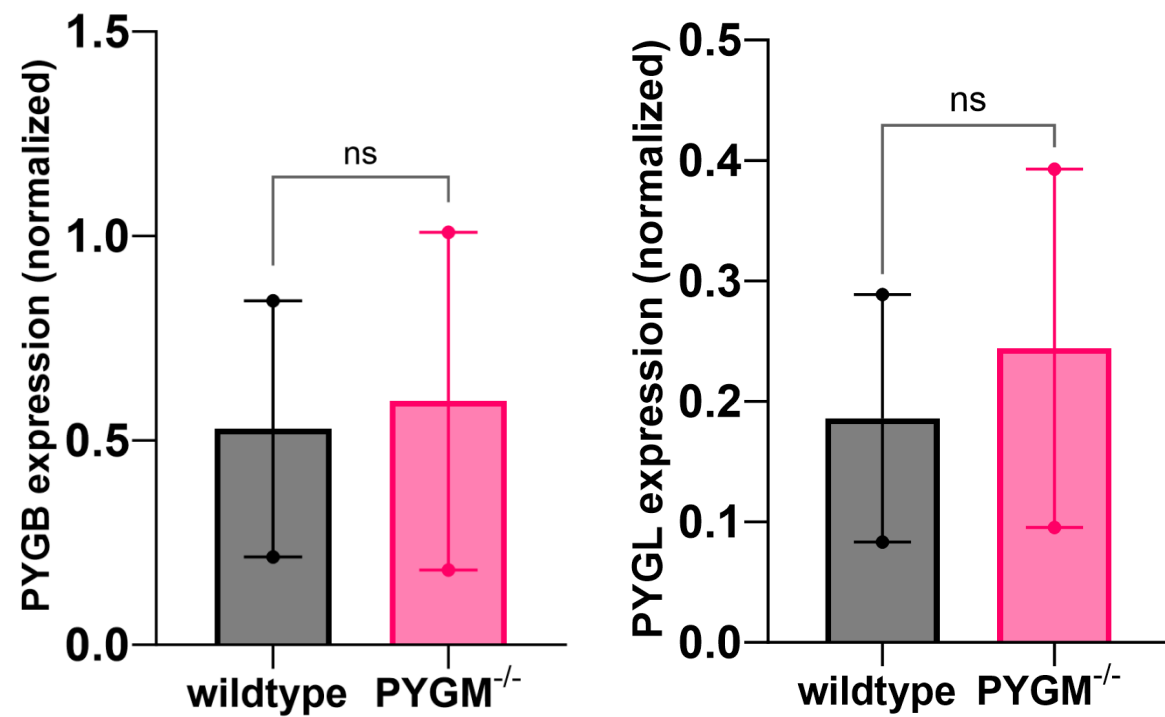

Supplement: Figure S1 [file mmc1.pdf]

**A**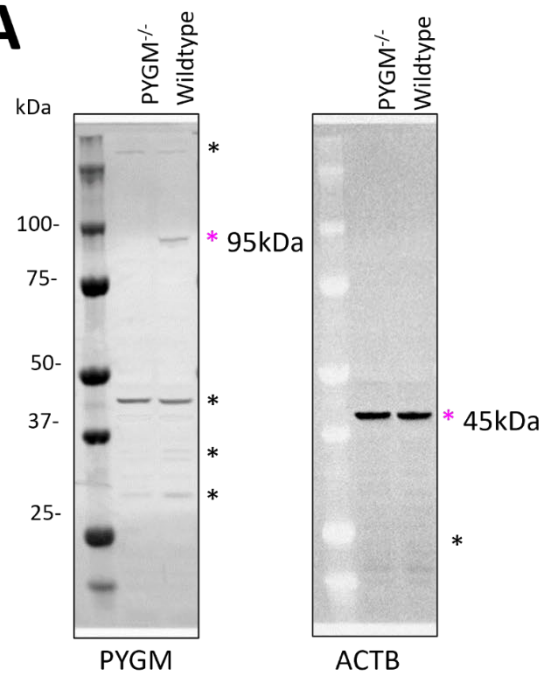**B**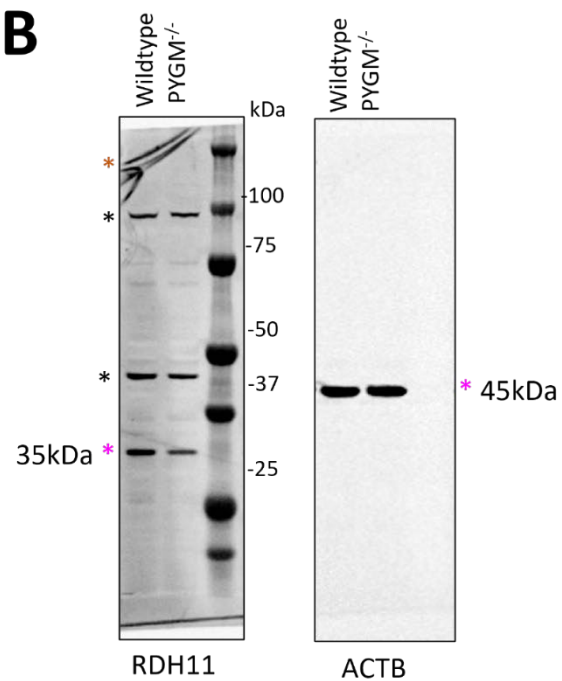

Supplement: Figure S2 [file mmc2.pdf]
