# Supplementary material for: Glycogen myophosphorylase loss causes increased dependence on glucose in iPSC-derived retinal pigment epithelium
Source: J Biol Chem. 2024 Jul 14;300(8):107569. doi: 10.1016/j.jbc.2024.107569 (PMC11342771; doi:10.1016/j.jbc.2024.107569)
Supplement: Supporting information [file mmc3.docx]

**Glycogen myophosphorylase loss causes increased dependence on glucose in iPSC-derived retinal pigment epithelium**

Basudha Basu, Magdalena Karwatka, Becky China, Martin McKibbin, Kamron Khan, Chris Inglehearn, John Ladbury, Colin A. Johnson

**Figure S1. Expression levels of PYGB and PYGL in the wildtype and mutant RPE cells.** (A) RT-PCR of wildtype and PYGM^-/-^ RPE for PYGB and PYGL. WT=wildtype RPE, mut= PYGM^-/-^ RPE, -ve=negative control and L=ladder. Representative images are shown here. (B) Quantitative analysis of RT-PCR bands normalized to GAPDH. (GAPDH RT-PCR images are reused from Figure 4F because these samples also served as loading controls for the RT-PCRs presented in Figure 4. All of these RT-PCR samples were run concurrently as part of the same experiment.) Ratio paired t-test was used for statistical analysis of different runs. Error bars represent S.E.M. *p < 0.05, **p < 0.01, ***p < 0.001, # p < 0.0001

**Figure S2. Immunoblots for PYGM and RDH11.** (A) Complete immmunoblot of wildtype and PYGM^-/-^ RPE against PYGM protein and loading control ACTB (B) Complete immmunoblot of wildtype and PYGM^-/-^ RPE against RDH11 protein and loading control ACTB. Pink asterisks mark the expected band size for these proteins. The black asterisks mark non-specific bands. The brown asterisk indicates a crease in the blot. PYGM is 95kDa, RDH11 is 35kDa and ACTB is 45kDa.

**Table S1**. Primer sequences used to assess gene expression levels by RT-PCR

**Table S2. Genes examined for off-target hits.** The off-target hit “score” is a function of the probability of hit. The score for PYGM would be 100 for the guide that was used. The primers listed were used to sequence for any off-target mutations. None of the genes were found to be mutated.

**Table S3**. List of all the antibodies used in this study along with sources and dilutions used.

**Table S4.** Settings used for LCMS analysis by Compound Discoverer 3.3.1.111
